# Characterization of the Link between Ornithine, Arginine, Polyamine and Siderophore Metabolism in *Aspergillus fumigatus*

**DOI:** 10.1371/journal.pone.0067426

**Published:** 2013-06-18

**Authors:** Nicola Beckmann, Lukas Schafferer, Markus Schrettl, Ulrike Binder, Heribert Talasz, Herbert Lindner, Hubertus Haas

**Affiliations:** 1 Division of Molecular Biology/Biocenter, Innsbruck Medical University, Innsbruck, Austria; 2 Division of Hygiene and Medical Microbiology, Innsbruck Medical University, Innsbruck, Austria; 3 Division of Clinical Biochemistry/Biocenter; Innsbruck Medical University, Innsbruck, Austria; Universidade de Sao Paulo, Brazil; Goldman

## Abstract

The opportunistic fungal pathogen *Aspergillus fumigatus* produces siderophores for uptake and storage of iron, which is essential for its virulence. The main precursor of siderophore biosynthesis (SB), ornithine, can be produced from glutamate in the mitochondria or by cytosolic hydrolysis of ornithine-derived arginine. Here, we studied the impact of mitochondrial versus cytosolic ornithine biosynthesis on SB by comparison of the arginine auxotrophic mutants *ΔargEF* and *ΔargB*, which lack and possess mitochondrial ornithine production, respectively. Deficiency in *argEF* (encoding acetylglutamate kinase and acetylglutamyl-phosphate-reductase), but not *argB* (encoding ornithine transcarbamoyl transferase) decreased (i) the cellular ornithine content, (ii) extra- and intracellular SB, (iii) growth under harsh iron starvation, (iv) resistance to the ornithine decarboxylase inhibitor eflornithine, and (v) virulence in the *Galleria mellonella* larvae model. These lines of evidence indicate that SB is mainly fueled by mitochondrial rather than cytosolic ornithine production and underline the role of SB in virulence. Ornithine content and SB of *ΔargB* increased with declining arginine supplementation indicating feedback-inhibition of mitochondrial ornithine biosynthesis by arginine. In contrast to SB, the arginine and polyamine contents were only mildly affected in *ΔargEF*, indicating prioritization of the latter two ornithine-consuming pathways over SB. These data highlight the metabolic differences between the two arginine auxotrophic mutants *ΔargEF* and *ΔargB* and demonstrate that supplementation of an auxotrophic mutant does not restore the wild type metabolism at the molecular level, a fact to be considered when working with auxotrophic mutants. Moreover, cross pathway control-mediating CpcA was found to influence the ornithine pool as well as biosynthesis of siderophores and polyamines.

## Introduction

Iron is able to adopt two ionic forms, reduced ferrous (Fe^2+^) or oxidized ferric (Fe^3+^), which in turn enables its contribution to various oxidation/reduction processes. Therefore it represents an essential nutrient for virtually every organism. Although highly abundant in the earth's crust, the bioavailability of iron is very low because oxyhydroxide colloid particles, the major oxidized form found in aerobic environments, display a solubility below 10^−9^ M at neutral pH, which is insufficient to sustain vital processes [1]. Apart from its crucial roles in respiration, oxidative stress detoxification, as well as biosynthesis of amino acids, sterols, and desoxyribonucleic acid, iron is able to generate toxic reactive species if accumulated excessively [2]. Hence, organisms had to develop fine-tuned regulatory mechanisms regarding uptake, storage and consumption of iron.

The ascomycete *Aspergillus fumigatus* is a typical saprophyte usually found in soil and on decaying organic matter. Nevertheless, this mold has become the most common airborne, pathogenic fungus causing life-threatening disease especially in immune-compromised patients [3]. In order to assure sufficient iron supply *A. fumigatus* employs a low-affinity and two high-affinity iron uptake systems, namely reductive iron assimilation and siderophore-assisted mobilization of iron [4], [5]. In reductive iron assimilation, cell-surface metalloreductases such as FreB reduce Fe^3+^ to Fe^2+^, which is then taken up by the low-affinity system or the high-affinity ferroxidase/iron permease (FetC/FtrA) complex [6]. Siderophore-assisted mobilization of iron involves production and excretion of the two siderophores, fusarinine C (FsC) and triacetylfusarinine C (TAFC) [7], [8]. Upon chelation of iron, siderophore-iron complexes are utilized either by reductive iron assimilation or are taken up by specific transporters [9], [10]. Additionally, *A. fumigatus* employs two intracellular siderophores, ferricrocin (FC) and hydroxyferricrocin (HFC), for hyphal iron distribution as well as hyphal and conidial iron storage [7], [11]. Extra- and intracellular siderophores represent crucial virulence determinants of *A. fumigatus* in different animal host systems as well as of phytopathogenic fungal species [4], [7], [12]–[15].

**Figure 1:**
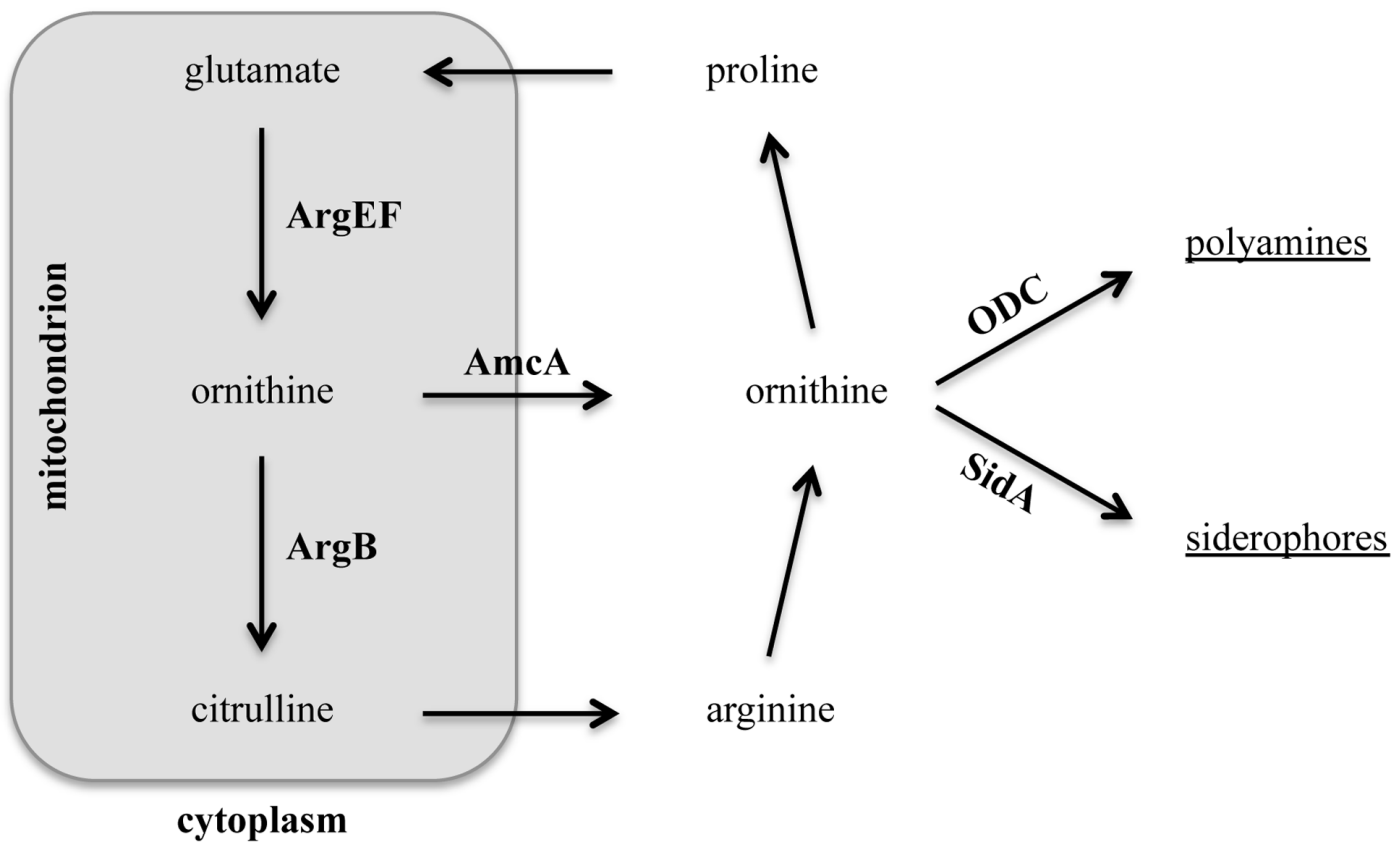
Schematic view of the metabolic link between ornithine, arginine, polyamines and siderophores in *A. fumigatus*. ArgEF: acetylglutamate kinase, acetylglutamyl-phosphate-reductase (AFUA_6g02910); ArgB: ornithine transcarbamoyl transferase (Afu4g07190); AmcA: mitochondrial ornithine exporter (AFUA_8g02760); ODC: ornithine decarboxylase (AFUA_4g08010); SidA: L-ornithine N5-oxygenase (AFUA_2g07680).

FsC is made up of three *N^5^*-*cis*-anhydromevalonyl-*N^5^*-hydroxyornithine residues, which are cyclically linked by ester bonds [8]. TAFC derives from FsC by *N^2^*-acetylation. FC is a cyclic hexapeptide, (*N^5^*-acetyl-*N^5^*-hydroxyornthine)_3_-Gly-Ser-Gly, and its hydroxylation leads to formation of HFC [8]. The major precursor of all of these siderophores is the non-proteinogenic amino acid ornithine [4], [16]. Ornithine is produced in mitochondria from glutamate involving six enzymes (Figure 1): acetylglutamate synthase, acetylglutamate kinase and acetylglutamyl-phosphate-reductase that are encoded by a single gene termed *argEF* in *A. fumigatus* (ortholog of *Saccharomyces cerevisiae ARG5,6*), acetylornithine-aminotransferase, acetylornithine-deacetylase, and arginine biosynthesis bi-functional enzyme [17], [18]. The acetylglutamate kinase is believed to be the rate-limiting step in arginine biosynthesis [19]. The produced ornithine is either exported into the cytosol by AmcA (ortholog of *S. cerevisiae* Arg11) or converted into citrulline by the ornithine transcarbamoyl transferase termed ArgB in *A. fumigatus* [20]. Citrulline is shuttled to the cytosol and converted into arginine via three steps. Arginine can be recycled into ornithine by arginase and back to glutamate via proline [18]. Availability of cytosolic ornithine is required for biosynthesis of siderophores and polyamines.

Reductive iron assimilation and siderophore biosynthesis (SB) are upregulated during iron starvation [21]. Moreover, iron starvation has been shown to dramatically impact the free amino acid pool of *A. fumigatus* including an increase of the cellular contents in ornithine and arginine by 7-fold and 11-fold, respectively [22]. Blocking ornithine consumption by SB via inactivation of the ornithine-*N^5^*-monooxygenase SidA, the first committed step in SB, increased the cellular ornithine content another 4-fold [22]. These data suggested that the increased ornithine demand for SB under iron deficiency is supplied by active up-regulation of ornithine biosynthesis rather than by de-repression of it via its consumption, as the latter would decrease the intracellular ornithine pool. Consistently, enzymes involved in biosynthesis of ornithine and arginine are transcriptionally upregulated during iron starvation [22].

Transcriptional repression of SB during iron sufficiency is mediated by the iron-responsive GATA transcription factor SreA [21]. The bZip-like transcription factor HapX is required for transcriptional activation of SB during iron starvation [22]. Deficiency in HapX, but not SreA has been shown to dramatically impact the free amino acid pool during iron starvation including a 10-fold decrease in ornithine, which might be responsible, at least in part, for the decreased SB in the mutant lacking HapX [22]. Interestingly, neither HapX nor SreA are required for the transcriptional upregulation of ornithine biosynthetic genes during iron starvation [22]. Furthermore, the sterol regulatory element binding protein SrbA was shown to be required for activation of SB during iron starvation [23].

In the current study, we characterized in detail the role of mitochondrial versus cytosolic ornithine supply in biosynthesis of siderophores and polyamines as well as in virulence by comparison of *argEF* and *argB* mutants. Moreover, we analyzed the roles of the cross pathway control-mediating transcription factor CpcA in biosynthesis of ornithine, polyamines and siderophores.

## Materials and Methods

### Fungal strains and growth conditions

*A. fumigatus* strains were cultivated at 37°C in *Aspergillus* minimal medium according to Pontecorvo et al. [24] containing 1% glucose as carbon and 20 mM glutamine as nitrogen source. For iron sufficiency, FeSO_4_ was added to a final concentration of 30 µM, for iron depleted conditions iron was omitted. Bathophenathroline disulfonate (BPS) was added to a concentration of 200 µM. For growth assays, 10^5^ spores were point-inoculated on plates and incubated at 37°C for 48 h. Liquid cultures were inoculated with 10^8^ spores/100ml medium. Fungal strains used in this study, are listed in Table S1.

### Analysis of siderophores, free amino acids and polyamines

Analysis of the free amino acid content was obtained by ethanol extraction and subsequent reversed phase HPLC as described before [25]. Intra- and extracellular siderophore were analyzed from culture supernatants as described previously [22]. For quantification of polyamines, 50 mg freeze-dried mycelium were homogenized and incubated with 6% perchloric acid for 3 h. Polyamine derivatization was carried out according to Wongyai et al. [26] with slight modifications. For preparation of the spermidine standard solutions, spermidine trihydrochloride (Sigma Aldrich) was dissolved in milliQ water to yield a stock solution of 200 nmol/ml. 2 M NaOH were added to 1 ml standard solution and perchloric acid-treated samples, respectively to reach a pH≥10. The volume was adjusted to 2 ml with milliQ water and 5 µl benzoyl chloride were added. The mixtures were vortexed immediately for 3 min and then rotated at 30 rpm for 20 min at RT. The reaction was terminated by addition of 2 ml saturated sodium chloride solution. All extractions were done twice by adding 1.5 ml diethyl ether, vortex-mixing for 3 min and rotating at 30 rpm for 20 min. The organic phases were withdrawn and pooled for each sample and standard, respectively and evaporated to dryness using a SpeedVac concentrator. The remaining residue was redissolved in 300 µl methanol followed by 200 µl milliQ water. Standard solutions were mixed and diluted with 60% methanol to produce concentrations of 50, 12.5 and 2.5 nmol/ml. Separation of benzoylated amines was carried out with isocratic reversed-phase HPLC using a Beckman 127 solvent module and a Nucleosil C_18_ column (250×4.0 mm, 100–5, Macherey-Nagel). 20 µl of each sample and standard solution were injected using a 20 µl Rheodyne 7725i loop injector. The mobile phase was methanol-water (60:40, v/v) and delivered at a flow rate of 0.5 ml/min. The amine derivatives were detected by means of an UV/Vis detector (SpectroMonitor 3200) at 234 nm. The detector signal was integrated and quantified using the Gold System (Beckman).

### DNA, RNA isolation and Northern analysis

Genomic DNA was extracted from homogenized mycelia according to Sambrook et al. [27]. RNA was isolated using TRI Reagent (Sigma) and peqGOLD Phase Trap (peqlab) reaction tubes. 10 µg of total RNA were analyzed as described previously [28]. Hybridization probes were amplified by PCR using the primers listed in Table S3.

### Deletion of *argEF* (AFUA_6g02910) and reconstitution of the *ΔargEF* strain

For generation of a *ΔargEF* mutant strain, the bipartite marker technique was used [29]. Briefly, *A. fumigatus* strain Cea17*-ΔakuB* was co-transformed with two DNA constructs, each containing an incomplete fragment of a pyrithiamine resistance gene (*ptrA*) [29] fused to 1.3 kb, and 1.3 kb of *argEF* flanking sequences, respectively. These marker fragments shared a 557 bp overlap within the *ptrA* cassette, which served as a potential recombination site. During transformation, homologous integration of each fragment into the genome flanking *argEF* allows recombination of the *ptrA* fragments and generation of the intact resistance gene at the site of recombination. Two rounds of PCR generated each fragment. First, each flanking region was amplified from ATCC 46645 genomic DNA using primer oargEF-1 and oargEF-4 for flanking region A (1.3 kb), and oargEF-2 and oargEF-3 for flanking region B (1.3 kb). Subsequent to gel-purification, the fragments were digested with *Xba*I and *Hind*III, respectively. The *ptrA* selection marker was released from plasmid pSK275 (gift from Sven Krappmann, Goettingen, Germany) by digestion with *Xba*I and *Hind*III, and ligated with the two flanking regions A and B described above. For generation of *ΔargEF*, two overlapping fragments were amplified from the ligation products using primers oargEF-5 and optrA-2 for fragment C (2.8 kb) and primers oargEF-6 and optrA-1 for fragment D (2.2 kb). Subsequently *ΔakuB* was transformed simultaneously with the overlapping fragments C and D. In the generated mutant allele of *ΔargEF-ptrA* the deleted region comprises amino acids 1 – 836 out of 906 in ArgEF.

For reconstitution of the *ΔargEF* strain with a functional *argEF* copy, a 4.9-kb PCR fragment, amplified using primers oargEF-5 and oargEF-6, was subcloned into pCR2.1-TOPO (Invitrogen). The resulting plasmid p*argEF* was linearized with *Ssp*I and used to transform *A. fumigatus ΔargEF* protoplasts. Taking advantage of the arginine auxotrophy of the *ΔargEF* mutant, protoplasts were transformed with p*argEF* and screened for wild-type growth in the absence of exogenous arginine for genetic complementation. Positive deletion- and reconstituted- strains were screened by Southern analysis and hybridization probes were generated using primers oargEF-5 and oargEF-4 (data not shown). Primers are listed in Table S2.

*A. fumigatus* transformation was carried out according to Tilburn et al. [30]. In order to obtain homokaryotic transformants, colonies from single homokaryotic spores were picked and single genomic integration was confirmed by PCR (data not shown) and Southern blot analysis.

### Eflornithine inhibition assay

Growth inhibitory effects of eflornithine (Sigma-Aldrich) were tested by agar diffusion assays. Therefore, 2×10^5^ conidia/ml were inoculated into 5 ml top agar and poured on agar plates containing 200 µM BPS or 30 µM FeSO_4_ and 5 mM arginine to enable growth of *ΔargEF* and *ΔargB*. For -Fe plates addition of FeSO_4_ was omitted. 100 µl of a 0.3 M eflornithine solution were added into a hole pricked in the middle of the plates. Fungal growth inhibition was scored after incubation for 48 h at 37°C.

### *Galleria mellonella* infection studies virulence assays

*G. mellonella* virulence testing was carried out according to Fallon et. al [31]. Sixth-instar larvae of

*G. mellonella* (Handler, Lebendköder-Großhandel, Leobersdorf, Austria) were stored in the dark at 18°C prior to use. Larvae weighing between 0.3 and 0.4 g were used, each (n = 20) infected with 1×10^7^ *A. fumigatus* conidia in 20 µl insect physiological saline (IPS) by injection into the hemocoel via the hind pro-leg. Freshly harvested conidial suspensions were filtered through a 40 µm nylon cell strainer (BD Falcon) before use. Untreated larvae and larvae injected with 20 µl of IPS served as controls. Larvae were incubated at 30°C in the dark and monitored daily up to 6 days. Three experiments were performed for each strain. Significance of mortality rate data was evaluated by using Kaplan-Meier survival curves with the PRISM statistics software (Mantel-Cox log rank test).

## Results and Discussion

### Mitochondrial ornithine production is crucial for adaptation to iron starvation

**Figure 2:**
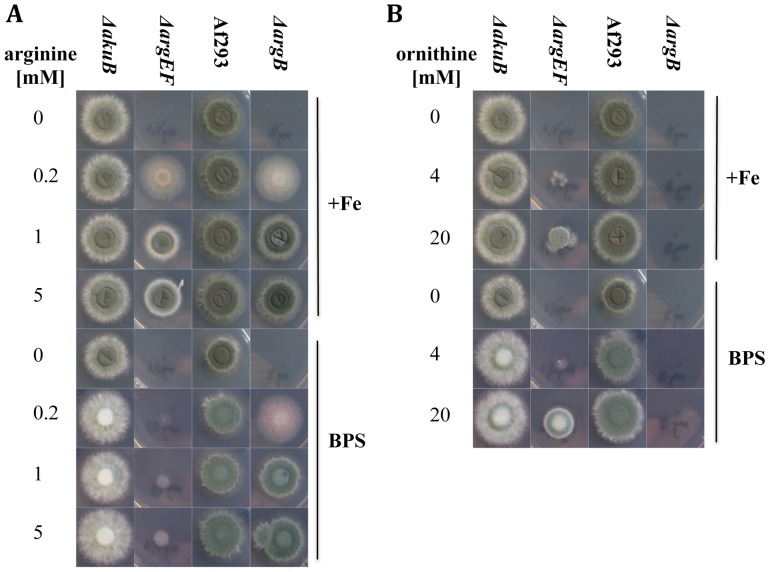
Inactivation of ArgEF but not ArgB impairs growth in the presence of BPS. Fungal growth on solid media was scored after 48 h on plates containing 0, 0.2, 1 or 5 mM arginine (A) as well as 0, 4, or 20 mM ornithine (B). The media were either iron depleted with 0.2 mM BPS or iron-replete (+Fe).

Previous studies showed, that iron deficiency causes an active upregulation of ornithine biosynthesis [22]. Ornithine is produced in mitochondria from glutamate or in the cytosol from ornithine-derived arginine [18]. To investigate the consequences of mitochondrial versus cytosolic ornithine production, especially under iron limited conditions, we compared two arginine auxotrophic mutant strains, *ΔargEF* and *ΔargB* and their respective wild type (wt) strains. *ΔargEF* was generated during this study in the *A. fumigatus ΔakuB* strain as described in *Material and Methods*. The *ΔakuB* strain is a derivative of *A. fumigatus* CEA17 lacking *akuB*, which facilitates gene deletion [32]. Generation of the *ΔargB* mutant strain in *A. fumigatus* Af293, kindly supplied by Dr. Nir Osherov, was described previously [20]. Important to note, reconstituted *ΔargEF* and *ΔargB* mutants, termed *argEF^c^* and *argB^c^*, respectively, generated by re-integration of wt gene copies showed wt-like phenotypes in all assays performed (growth, SB, virulence) emphasizing the specificity of the gene deletion phenotypes (data not shown). The growth medium used, containing glutamine as nitrogen source, was additionally supplemented with arginine to enable growth of the auxotrophic mutants. Under these conditions, both mutant strains are capable of producing ornithine by cytosolic hydrolysis of arginine (Figure 1). In contrast to *ΔargEF*, *ΔargB* is able to produce ornithine from glutamine-derived glutamate in the mitochondria (Figure 1). The growth of the mutant strains was compared to the respective genetic wt strains by spotting 10^5^ conidia on solid minimal medium representing iron-replete and depleted conditions containing different concentrations of arginine (0, 0.2 mM, 1 mM, 5 mM) (Figure 2A). As expected, inactivation of ArgEF as well as ArgB resulted in arginine auxotrophy (Figure 2A). During iron sufficiency (30 µM FeSO_4_), 0.2 mM arginine rescued growth but not sporulation of both mutant strains. To restore sporulation concentrations of 1 mM arginine and above were required. Blocking reductive iron assimilation with the ferrous iron-specific chelator BPS, which renders the siderophore system the only functional high-affinity iron assimilation system [4], severely impaired growth of *ΔargEF* but not *ΔargB*. These data indicate that inactivation of ArgEF but not ArgB impairs adaptation to iron starvation, most likely due to the crucial role of mitochondrial ornithine production for SB.

Supplementation with ornithine instead of arginine rescued growth of *ΔargEF* only partially, possibly due to inefficient cellular ornithine uptake or insufficient transport of ornithine into mitochondria and therefore inefficient arginine synthesis from the added ornithine (Figure 2B). In contrast to arginine supplementation, the radial growth of ornithine-supplemented *ΔargEF* was not decreased by BPS, which again underlines the importance of mitochondrial ornithine production for SB. Consistent with the biosynthetic pathway depicted in Figure 1, *ΔargB* is unable to synthesize arginine from ornithine and therefore to grow with ornithine supplementation (Figure 2B).

### ArgEF-deficiency decreases, while ArgB-deficiency increases SB and the intracellular ornithine pool

The influence of mitochondrial ornithine production on SB was examined by measurement of extra- and intracellular siderophores of *ΔargEF* and *ΔargB* in iron deficient liquid cultures supplemented with 0.2 mM, 1 mM or 5 mM arginine (Table 1). Consistent with impaired SB being responsible for the defective growth of *ΔargEF* in the presence of BPS (Figure 2A), deficiency in ArgEF, but not ArgB, decreased production of both extra- and intracellular siderophores compared to the respective wt strains. In more detail, declining arginine availability decreased extra- and intracellular SB of *ΔargEF*, e.g., extracellular SB of *ΔargEF* was reduced by 64% with 5 mM arginine supplementation and by 97% with 0.2 mM arginine supplementation (Table 1). In contrast, declining arginine availability elevated extra- and intracellular SB of *ΔargB*, e.g. extracellular SB of *ΔargB* was decreased by 16% with 5 mM arginine supplementation but 1.6-fold increased with 0.2 mM arginine supplementation (Table 1).

**Table 1:** Deficiency in ArgEF but not ArgB decreases extra- and intracellular siderophore production.

arginine [mM]	strain	siderophores [%]	
		intracellular	extracellular
**5**	***ΔargEF***	39.69±4.83	35.84±3.68
	***ΔakuB***	**100.00**	**100.00**
	***ΔargB***	111.23±19.38	83.68±12.20
	**Af293**	**100.00**	**100.00**
**1**	***ΔargEF***	12.91±2.86	5.04±0.91
	***ΔakuB***	121.92±12.89	119.77±14.16
	***ΔargB***	149.64±23.17	126.46±19.13
	**Af293**	112.39±16.18	106.42±17.77
**0.2**	**Δ** ***argEF***	3.96±1.81	3.31±1.54
	**Δ** ***akuB***	109.10±10.31	103.11±3.08
	**Δ** ***argB***	190.79±17.71	202.13±13.90
	**Af293**	132.54±25.34	123.72±20.17
	**Δ** ***cpcA***	79.6±2.18	83.86±3.64
	**D141**	**100.00**	**100.00**

Siderophore production was quantified in 24 h liquid cultures representing iron starvation. For *ΔargEF*, *ΔargB* and their wt strains, the analysis was performed with 0.2, 1 and 5 mM arginine supplementation, respectively. *ΔcpcA* and its wt strain were analyzed without arginine supplementation. The data of mutant strains were normalized to that of the respective wt, in case of the *ΔargEF* and *ΔargB* mutants to the 5 mM arginine values. The data represent the mean of three biological replicates ± standard deviation.

To analyze the effects of deficiency in ArgB or ArgEF on amino acid metabolism, the free amino acid pools of both mutants and respective wt strains were analyzed in iron-starved liquid cultures with 1 mM arginine supplementation and additionally with 5 mM arginine for *ΔargEF* and its wt as well as with 0.2 mM for *ΔargB* and its wt (Tables S4 and 2). Table 2 displays the differences in free amino acid pools between the mutants compared to the respective wt strains. ArgEF-deficiency decreased the intracellular ornithine level by 85% with 5 mM arginine and by 98% with 1 mM arginine supplementation, while the arginine content remained about wt-like (Table 2). This dramatically reduced cellular ornithine content in *ΔargEF* agrees with the defect in SB (Table 1). ArgB-deficiency increased the cellular ornithine content, 1.6-fold with 1 mM and 3.1-fold with 0.2 mM arginine addition (Table 2), which is most likely the reason for the elevated SB of *ΔargB* under low arginine supply (Table 1).

**Table 2:** Deficiency in ArgEF or CpcA but not ArgB decreases the cellular ornithine content.

	5 mM arginine	1 mM arginine	1 mM arginine	0.2 mM arginine	
**aa**	***ΔargEF/ΔakuB***	***ΔargEF/ΔakuB***	***ΔargB*** **/Af293**	***ΔargB*** **/Af293**	***ΔcpcA*** **/D141**
**Ala**	0.86	0.90	1.30	**1.51**	**0.52**
**Arg**	0.94	0.97	1.09	**0.56**	0.93
**Asn**	0.98	1.00	0.93	1.23	0.82
**Asp**	0.97	0.85	0.76	1.15	0.73
**Gln**	1.03	0.99	0.91	0.76	1.49
**Glu**	1.21	1.26	0.90	1.09	0.84
**Gly**	0.69	**0.60**	1.14	1.36	0.90
**His**	0.79	1.36	1.07	1.37	0.90
**Ile**	0.91	0.88	1.28	**1.93**	0.69
**Leu**	0.68	**0.59**	1.15	**1.72**	**0.58**
**Lys**	0.77	0.72	0.80	1.10	0.67
**Met**	**0.62**	0.75	0.85	**1.58**	0.86
**Orn**	***0.15***	***0.02***	**1.55**	***3.12***	**0.45**
**Phe**	0.82	0.73	1.23	**2.00**	**0.56**
**Ser**	0.99	0.97	0.92	1.33	0.82
**Thr**	1.00	1.07	0.99	1.48	**0.61**
**Trp**	**0.51**	**0.43**	1.31	**1.58**	**0.40**
**Tyr**	0.78	**0.63**	1.14	**2.61**	**0.59**
**Val**	0.78	0.79	1.41	**2.01**	**0.59**

*ΔakuB and ΔargEF* were supplemented either with 5 or 1 mM arginine; Af293 and *ΔargB* were supplemented either with 1 or 0.2 mM arginine. Individual amino acid pools are given in % of the total free amino acids and normalized to that of the respective wt. Amino acid pools up- or down-regulated >1.5- and >3-fold in mutant strain versus wt are in bold and *bold*, respectively. Given values represent the mean of three biological replicates, the standard deviations are given in Table S4.

Taken together, the defects in growth and SB as well as the decreased ornithine content observed during iron starvation in *ΔargEF* but not *ΔargB* indicate that SB is mainly fueled by mitochondrial rather than cytosolic ornithine production. Moreover, the increase in ornithine content (Table 2) and SB (Table 1) with declining arginine supplementation and decreased arginine content (Table 2) in *ΔargB* suggest that mitochondrial ornithine biosynthesis is feedback-inhibited by arginine. In agreement, N-acetylglutamate synthase, N-acetylglutamate kinase and N-acetylglutamylphosphate reductase (the latter two encoded by *argEF*) have been shown to be feedback inhibited by arginine in *N. crassa* [33], [34]. Notably, several other amino acids were affected by deficiency in either ArgB or ArgEF, although not as severely as ornithine (Table 2). These data underline the interconnection of amino acid metabolism and highlight the differences between these two arginine auxotrophic mutants at the molecular level. Moreover, the study demonstrates that supplementation of an auxotrophic mutant with the respective amino acid does not restore the wt metabolism at the molecular level.

### The cellular polyamine content is not affected by ArgB-deficiency, moderately decreased by ArgEF-deficiency, moderately increased by CpcA-deficiency, and largely upregulated by HapX-deficiency

Polyamines are essential for growth, cell proliferation and differentiation, but toxic in excess [35], [36]. The rate-limiting enzyme in polyamine biosynthesis is cytosolic ornithine decarboxylase that catalyzes the conversion of ornithine to the divalent polyamine putrescine, which is then converted by spermidine synthetase to spermidine, the major polyamine of *Aspergilli* [37]. To investigate the interplay between biosynthesis of ornithine, siderophores and polyamines, we analyzed the spermidine content of *ΔargEF*, *ΔargB* and their respective wt strains during iron-replete and iron depleted conditions with 1 mM arginine supplementation (Table 3), because the previous experiments showed that effects resulting from the gene deletion are best seen at this concentration (Tables 2 and 3). Iron starvation significantly reduced the cellular spermidine content in all wt strains analyzed, i.e. to 36%–55% of the content during iron sufficiency. The cellular spermidine pool was not affected by ArgB-deficiency and only moderately decreased by ArgEF-deficiency, i.e. by 27% during iron starvation and 16% during iron sufficiency. In other words, *A. fumigatus* maintains a constant polyamine level irrespective of decreased (98% decrease in *ΔargEF*) or increased (1.6-fold increased in *ΔargB*) cellular content of the polyamine precursor ornithine (Table 2).

**Table 3:** Impact of deficiency in ArgEF, ArgB, HapX and CpcA on the cellular spermidine content.

	spermidine nmol/mg dw		ratio mutant/wt
	*ΔakuB*	*ΔargEF*	
**+Fe**	10.66±0.00	8.93±1.31	**0.84**
**−Fe**	4.18±0.14	3.07±0.06	**0.73**
**ratio –Fe/+Fe**	**0.39**	**0.34**	
	**Af293**	***ΔargB***	
**+Fe**	9.66±0.62	11.17±2.26	**1.16**
**−Fe**	5.32±0.49	5.09±0.19	**0.96**
**ratio –Fe/+Fe**	**0.55**	**0.46**	
	**D141**	***ΔcpcA***	
**+Fe**	8.95±0.33	14.83±0.18	**1.66**
**−Fe**	3.82±0.07	5.29±0.13	**1.38**
**ratio –Fe/+Fe**	**0.43**	**0.36**	
	**wt**	***ΔhapX***	
**+Fe**	8.25±1.51	8.71±0.91	**1.06**
**−Fe**	2.95±0.46	17.39±1.02	**5.89**
**ratio –Fe/+Fe**	**0.36**	**2.00**	

Cultures of *ΔakuB, ΔargEF*, Af293 and *ΔargB* were supplemented with 1 mM arginine. The values are the mean ± standard deviation of three biological replicates.

Taken together, the dramatic reduction of ornithine content (Table 2) and SB (Table 1) paralleled by the unaltered arginine pool (Table 2) and only mildly decreased polyamine content (Table 3) in *ΔargEF* indicate prioritization of arginine and ornithine for cellular needs other than SB, e.g. arginine for protein biosynthesis and ornithine for polyamine biosynthesis. Another possibility could be the presence of an alternative and maybe compensatory polyamine biosynthetic pathway. Although most organisms produce polyamines exclusively from ornithine via ornithine decarboxylase, some bacteria, plants and cryptosporidia are capable of synthesizing putrescine also from arginine via arginine decarboxylase and agmatine ureohydrolase [38]. However, the existence of this pathway in *Ascomycetes* is unlikely as *S. cerevisiae*, *N. crassa* and *A. nidulans* strains carrying mutations in the ornithine decarboxylase gene are putrescine auxotrophic [38]–[41]. In further agreement, the growth defects of *A. fumigatus* caused by eflornithine-mediated inhibition of ornithine decarboxylase are cured by putrescine supplementation (data not shown).

Consistent with cellular balancing of SB and arginine metabolism, arginine was recently identified to allosterically activate the ornithine monooxygenase SidA and consequently SB-mediated ornithine consumption [42]. The rational for this regulation might be that *A. fumigatus* possesses reductive iron assimilation as an alternative ornithine-independent high-affinity iron acquisition system [4].

HapX-deficiency has previously been shown to strongly upregulate the ornithine decarboxylase transcript level during iron starvation [22]. In the current study, the spermidine content of the *ΔhapX* mutant was found to be wt-like during iron sufficiency (Table 3), which is consistent with HapX functioning solely under iron starvation as previously suggested [22]. In contrast, HapX-deficiency increased the spermidine content 5.9-fold during iron starvation. These data suggest that the transcriptional upregulation of ornithine decarboxylase most likely represents a deregulation and not a response to the decreased cellular ornithine content measured in *ΔhapX* [22]. Additional support is provided by the fact that similar to HapX-deficiency, lack of ArgEF decreases the cellular ornithine pool, but this is accompanied by a slight decrease in the spermidine pool, which contrasts the situation in *ΔhapX*. Consequently, increased transcription of ornithine decarboxylase and the resulting elevated polyamine pool might be partly responsible for the decrease in SB found in *ΔhapX* [22].

### Deficiency in ArgEF but not ArgB decreases resistance to the ornithine decarboxylase inhibitor eflornithine

**Figure 3:**
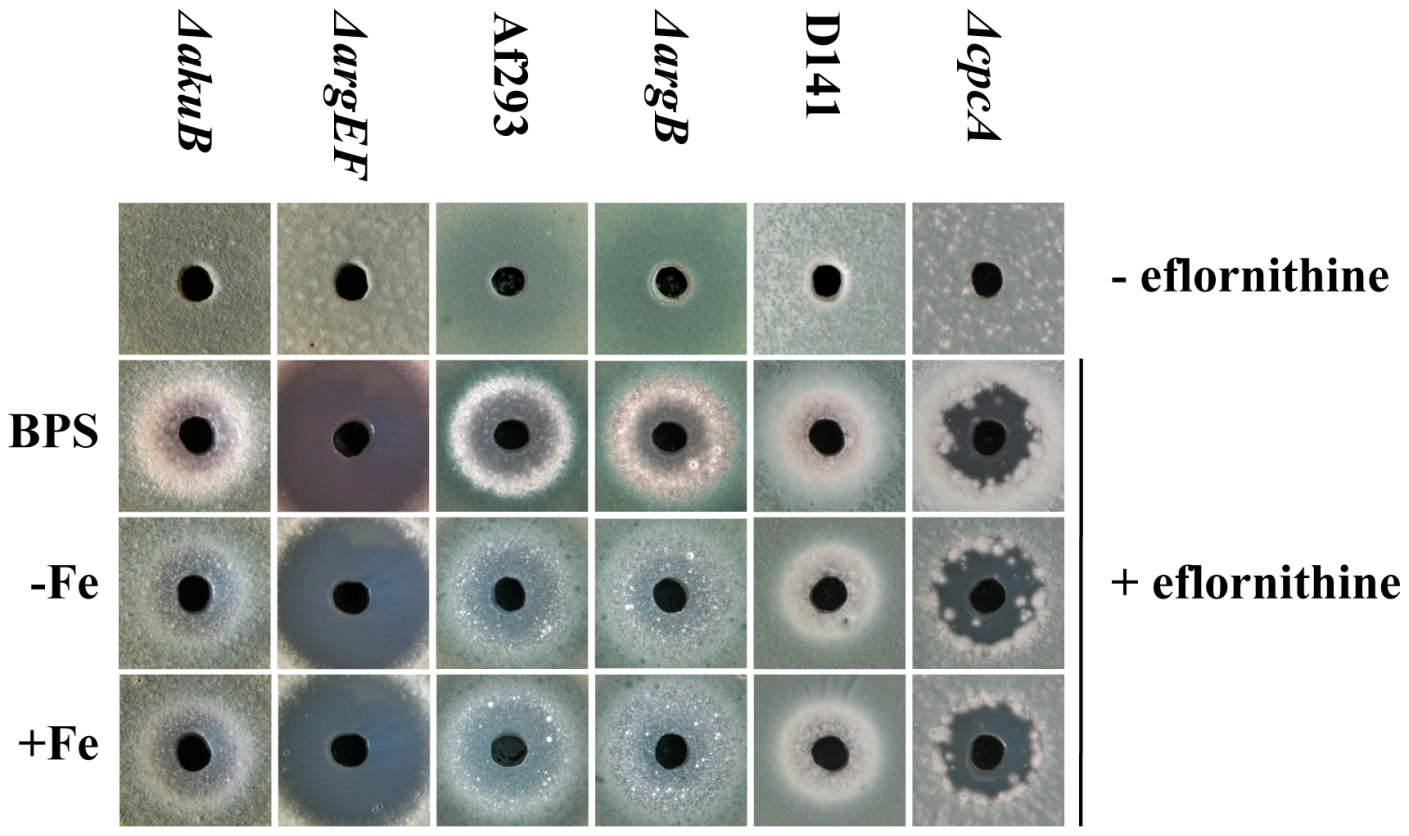
Deficiency in ArgEF but not ArgB increases susceptibility to eflornithine during iron starvation (BPS and −Fe) and iron sufficiency (+Fe). Inhibition of growth on solid media was scored after 48 h.

Polyamine biosynthesis can be blocked by eflornithine (α-difluoromethylornithine), an irreversible inhibitor of ornithine decarboxylase that is used in the treatment of hirsutism (excessive hair growth) as well as in African trypanosomiasis (sleeping sickness) [43], [44]. An agar diffusion assay demonstrated that *A. fumigatus* is susceptible to eflornithine, with similar growth inhibition during iron sufficiency and limitation (Figure 3). Compared to the respective wt, deficiency in ArgEF but not ArgB significantly increased susceptibility to eflornithine (Figure 3), which most likely reflects the dramatically decreased cellular ornithine content (Table 2) and the modestly decreased spermidine content (Table 3).

### ArgEF-deficiency causes transcriptional upregulation of genes involved in biosynthesis of ornithine/arginine and polyamines

**Figure 4:**
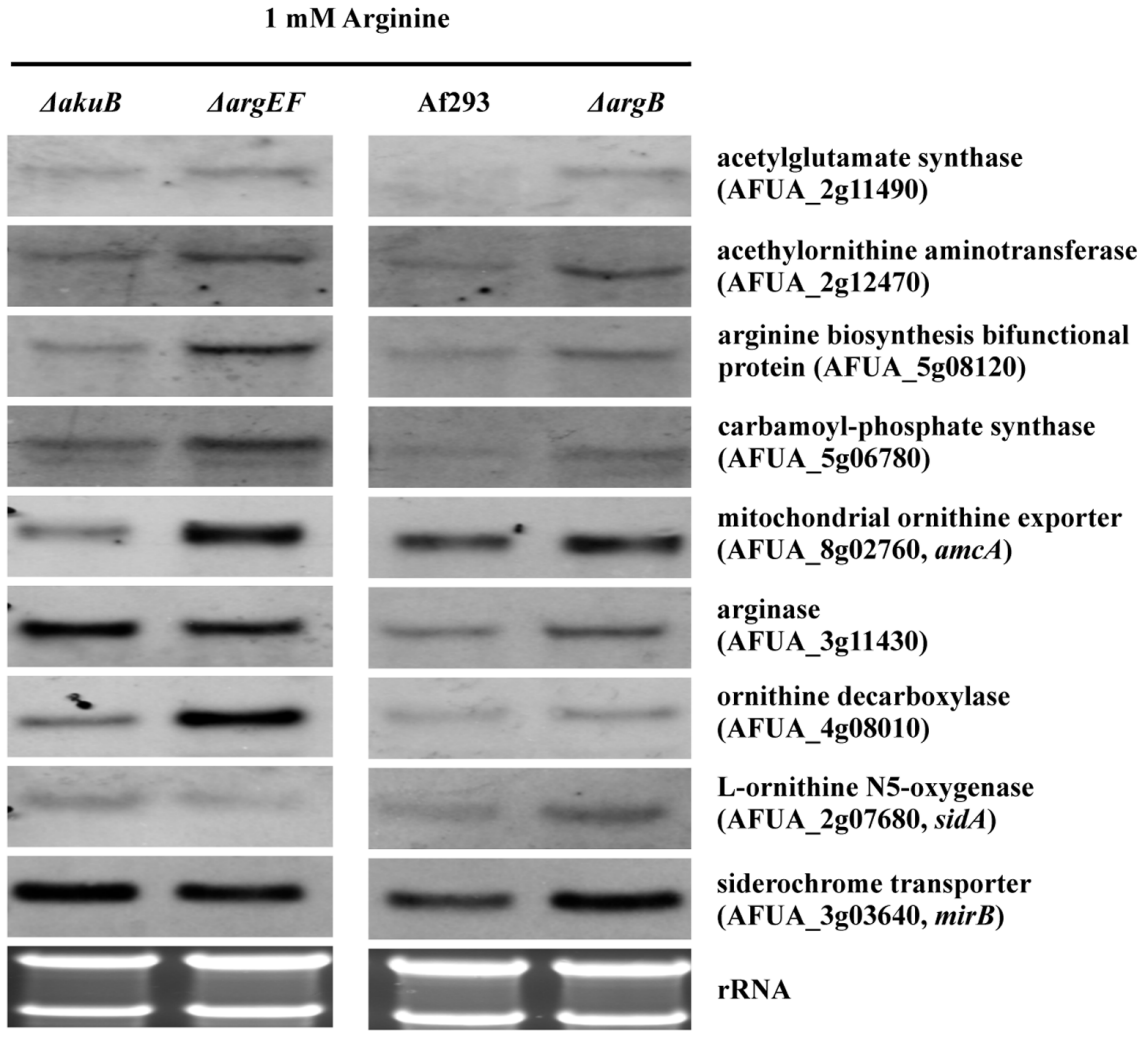
Deletion of ***argEF*** **leads to transcriptional upregulation of genes involved in ornithine biosynthesis, mitochondrial ornithine export and polyamine production.** Northern analysis was performed with RNA isolated from 24 h-liquid cultures representing iron starvation supplemented with 1 mM arginine.

To analyze the effects of deficiency in either ArgEF or ArgB on biosynthesis of ornithine/arginine and polyamines at the transcriptional level, Northern blot analysis of involved genes was performed from iron-starved cultures supplemented with 1 mM arginine (Figure 4). Compared to its wt, deficiency in ArgEF slightly upregulated transcript levels of the arginine biosynthesis bifunctional protein (AFUA_5g08120), the carbamoyl-phosphate synthase (AFUA_5g06780) and in particular the mitochondrial ornithine exporter AmcA (AFUA_8g02760). This upregulation might be caused by the disturbed ornithine/arginine metabolism or increased iron starvation of *ΔargEF*. The latter is unlikely as the iron-responsive genes *mirB* (AFUA_3g03640) and *sidA* (AFUA_2g07680) display equal transcript levels in *ΔargEF* and its respective wt strain. *mirB* and *sidA* encode a siderophore transporter and ornithine monooxygenase, which represents the first committed step in SB, respectively [4], [10], [21]. Therefore, these data also indicate that siderophore-metabolic genes do not transcriptionally respond to the cellular content of its precursor ornithine. ArgEF-deficiency also significantly upregulated the transcript level of ornithine decarboxylase (AFUA_4g08010), most likely to counteract the cellular decrease in ornithine to keep the polyamine level constant. Moreover, ArgEF-deficiency downregulated arginase (AFUA_3g11430). ArgB-deficiency modestly upregulated ornithine/arginine biosynthetic genes but not as much as ArgEF-deficiency.

### Deficiency in ArgEF but not ArgB attenuates virulence of *A. fumigatus* in an insect host model

**Figure 5:**
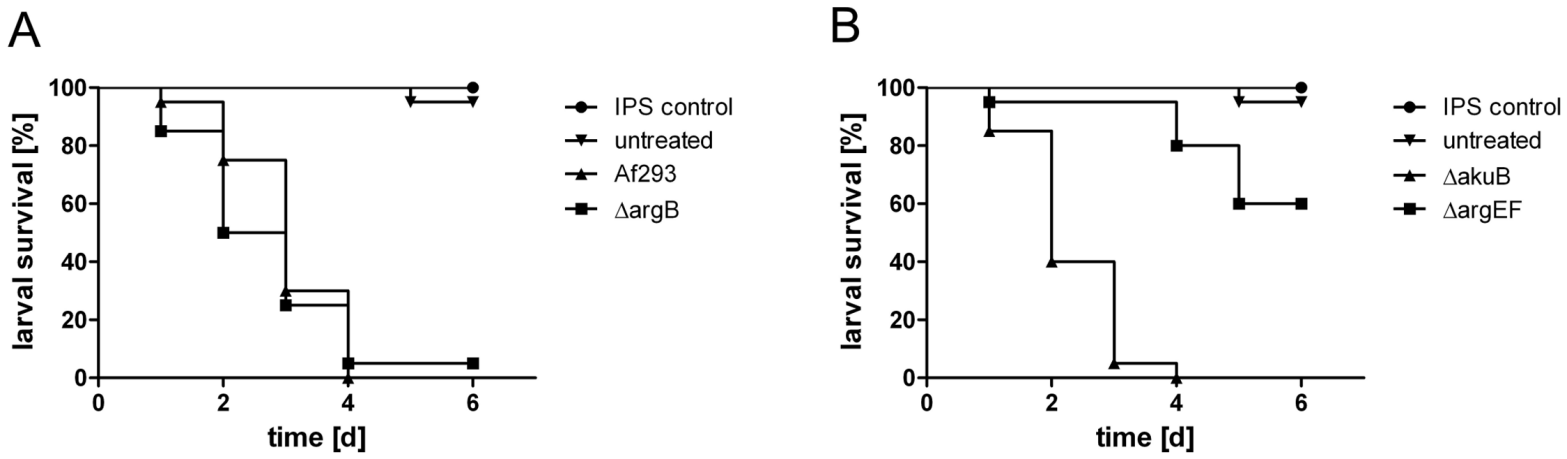
Deficiency in ArgEF but not ArgB attenuates virulence of *A. fumigatus* in the *Galleria mellonella* infection model. No significant difference in virulence between *ΔargB* and Af293 could be detected (P = 0.4423) (A). In contrast, larvae infected with the *ΔargEF* mutant strain exhibited significantly increased survival rates compared to larvae infected with the respective wt *ΔakuB* in this model (P < 0.0001) (B). The reconstituted *ΔargEF* strain, *argEF^c^*, displayed *ΔakuB*-like virulence (data not shown). Insect physiological saline (IPS) was used as an injection control. All larvae in this group remained viable for the entire experiment.

To assess the role of ornithine/arginine biosynthesis in pathogenicity of *A. fumigatus,* we compared Δ*argB* and Δ*argEF* mutants and their respective wt strains in the *G. mellonella* infection model [31]. The *argB* gene does not seem to play a primary role for virulence in *G. mellonella*, as disruption of the gene results in no significant difference in survival rates (P = 0.4423) over a period of 6 days (Figure 5A). In the first 48 h after infection, survival of larvae infected with the Δ*argB* mutant strain was reduced in comparison to those infected with Af293 (50% for Δ*argB* versus 75% for Af293), but 72 h after infection and at later time points, the difference in survival between the two strains was only 5%. The wt-like virulence of the arginine-auxotrophic Δ*argB* mutant indicates that arginine availability plays no limiting role for virulence of *A. fumigatus* in the insect model. Similarly, the histidine-auxotrophic mutant lacking homocitrate synthase (HcsA) was found to retain full virulence when injected intravenously in a murine model of invasive aspergillosis [45].

In contrast to Δ*argB*, the Δ*argEF* mutant exhibited attenuated virulence in the *G. mellonella* infection model, resulting in significantly higher survival compared to larvae infected with the Δ*akuB* strain (*P* < 0.001) (Figure 5B). At 24 h after infection 95% of the larvae infected with the Δ*argEF* strain remained alive, while 85% survived infection with Δ*akuB*. Already 48 h after infection, the attenuating effect of *argEF* disruption on virulence was more pronounced, resulting in survival rates of 95% in larvae infected with the Δ*argEF* mutant, and only 40% of survival in those infected with the Δ*akuB* strain. By 96 h, all of the larvae infected with the Δ*akuB* strain were dead, whereas still 80% of larvae infected with the Δ*argEF* mutant remained alive. Overall, survival proportions between larvae infected with the Δ*argEF* mutant strain or the Δ*akuB* strain are highly significant (*P* < 0.001), which shows that the loss of ArgEF leads to reduced virulence in the *G. mellonella* infection model. In comparison, the siderophore-lacking *ΔsidA* mutant, that has previously been demonstrated to be avirulent in both murine and *G. mellonella* infection models [4], [15], was virtually avirulent in the insect virulence assay (Figure S1) emphasizing the accuracy of this analysis.

As SB represents a virulence determinant for *A. fumigatus*, the virulence attenuation of Δ*argEF* in contrast to Δ*argB* might be caused by the dramatic decrease in SB seen in Δ*argEF* but not Δ*argB* (Table 1). Moreover, the disturbance of amino acid homeostasis observed in the amino acid pool analysis (Table 2) might contribute to the virulence defect of Δ*argEF*, as amino acid homeostasis is also critical for virulence of *A. fumigatus* [46].

### Biosynthesis of ornithine is affected by CpcA

**Figure 6:**
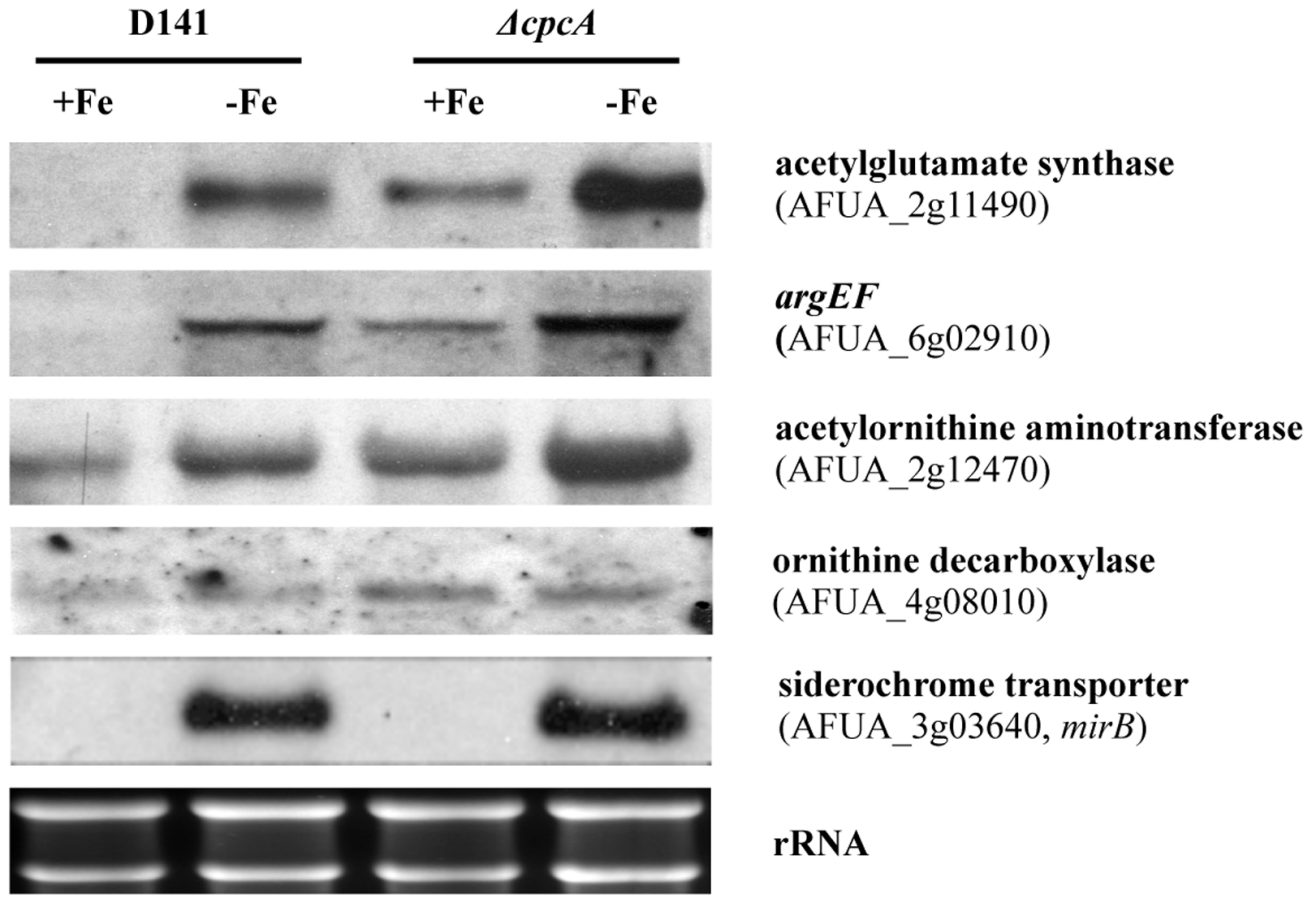
CpcA deficiency increases transcription of ornithine biosynthetic genes during iron starvation and more considerably under iron sufficiency. Northern analysis was performed with RNA isolated from 24 h-liquid cultures representing iron starvation (−Fe) and iron sufficiency (+Fe), respectively.

Previous data suggested that the increased demand of ornithine for SB under iron limitation is satisfied by transcriptional upregulation of its biosynthesis and not of its derepression via siderophore-mediated iron consumption [22]. The transcription factor HapX was found to be essential for the upregulation of the ornithine pool during iron starvation but transcriptional upregulation of ornithine/arginine biosynthetic genes was found to be independent of the iron regulators SreA and HapX [22]. These data raise the question of the responsible regulatory mechanism. Therefore, in a next approach we analyzed a potential involvement of the transcriptional activator CpcA, which mediates “cross pathway control” that is crucial for amino acid homeostasis and virulence of *A. fumigatus* [46]. The *ΔcpcA* mutant strain, which was generated in *A. fumigatus* strain D141 was described previously and kindly supplied by Dr. Sven Krappmann. In agreement with a decisive role in amino acid metabolism, analysis of the free amino acid pools during iron starvation revealed that CpcA-deficiency decreases the content of all amino acids with exception of glutamine (Tables 2 and S4). Remarkably, with a decrease to 45% of the wt content, ornithine was the second most affected amino acid in Δ*cpcA*. Consistent with a decisive role of the ornithine pool for SB, CpcA-deficiency decreased both extra- and intracellular SB to about 80% of the wt (Table 1). Northern analysis demonstrated transcriptional upregulation of the three ornithine/arginine biosynthetic genes encoding acetylglutamate synthase (AFUA_2g11490), ArgEF (AFUA_6g02910) and acetylornithine aminotransferase (AFUA_2g12470) in *A. fumigatus* D141 (Figure 6), as previously shown for strain ATCC 46645 [22]. CpcA-deficiency slightly upregulated the transcript levels of these genes during iron starvation (Figure 6), which might be a response to the decreased ornithine level (Table 2). Remarkably, CpcA-deficiency dramatically increased the transcript levels of these genes during iron-replete conditions (Figure 6), which raises the question whether expression of the ornithine/arginine biosynthetic genes is repressed during iron-replete conditions rather than activated during iron starvation. In *S. cerevisiae*, the CpcA ortholog Gcn4 was recently shown to mediate both transcriptional activation and repression [47]. Consistent with slight upregulation of ornithine decarboxylase transcript levels during both iron sufficiency and starvation (Figure 6), CpcA-deficiency also increased the cellular spermidine content (Table 3) with slightly increasing eflornithine susceptibility (Figure 3). The increase in spermidine might play a role in depletion of the ornithine pool and the decrease in SB.

### Conclusions

Taking advantage of arginine-auxotrophic mutant strains, the presented study characterized the link between metabolism of arginine, ornithine, polyamines and siderophores in *A. fumigatus*. The data indicate that (i) SB is fueled mainly by mitochondrial ornithine supply, (ii) mitochondrial ornithine production is feedback-inhibited by arginine, (iii) arginine and polyamine biosynthesis are prioritized over SB in ornithine supply, (iv) *A. fumigatus* is susceptible to the ornithine decarboxylase inhibitor eflornithine, (v) arginine biosynthesis does not play a major role in virulence of *A. fumigatus* in the insect host model, and (vi) supplementation of an auxotrophic mutant might not restore wt metabolism at the molecular level, which has to be considered when working with auxotrophic mutant.

## Supporting Information

Figure S1 **Deficiency in SidA, which blocks siderophore biosynthesis, attenuates virulence of** ***A. fumigatus*** **in the** ***Galleria mellonella*** **infection model.** Larvae infected with the *A. fumigatus ΔsidA* mutant strain exhibited significantly increased survival rates compared to larvae infected with *ΔakuB* in this model (P < 0.0001). Insect physiological saline (IPS) was used as an injection control, and all larvae in this group remained viable for the entire experiment.
(TIF)

Table S1 **Fungal strains used in this study.**
(DOC)

Table S2 **Primers used for generation of** ***ΔargEF and argEF^c^*** **.**
(DOC)

Table S3 **Primers used for amplification of hybridization probes.**
(DOC)

Table S4 **Free amino acid pools of** ***ΔargEF ΔargB*** **,** ***ΔcpcA*** **and the respective wt strains.** Individual amino acid pools are given in % of the total free amino acids.
(DOC)
