# Supplementary figures and images for: Characterization of the Link between Ornithine, Arginine, Polyamine and Siderophore Metabolism in Aspergillus fumigatus
Source: PLoS One. 2013 Jun 18;8(6):e67426. doi: 10.1371/journal.pone.0067426 (PMC3688985; doi:10.1371/journal.pone.0067426)

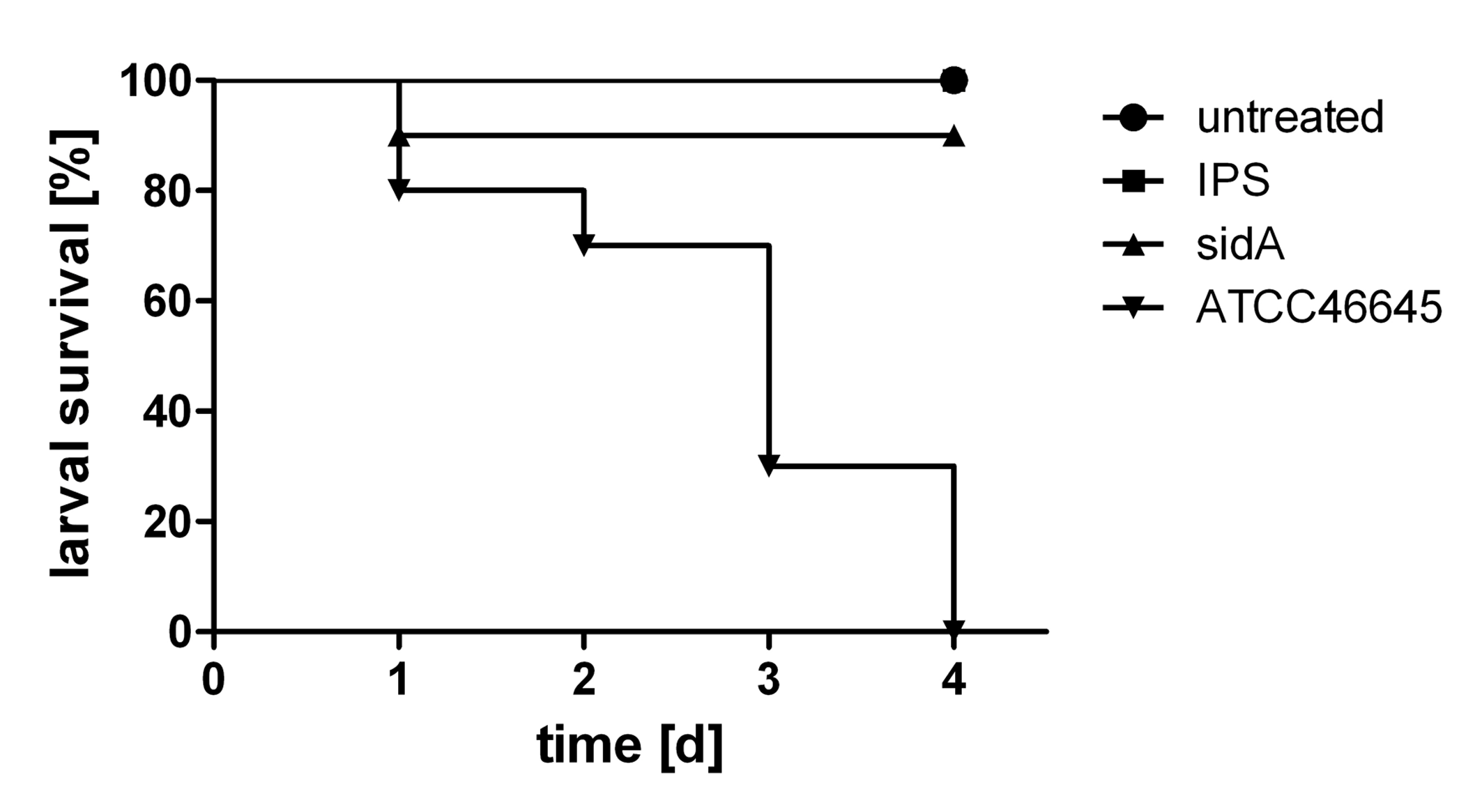

Supplement: Figure S1 — Deficiency in SidA, which blocks siderophore biosynthesis, attenuates virulence of A. fumigatus in the Galleria mellonella infection model. Larvae infected with the A. fumigatus ΔsidA mutant strain exhibited significantly increased survival rates compared to larvae infected with ΔakuB in this model (P < 0.0001). Insect physiological saline (IPS) was used as an injection control, and all larvae in this group remained viable for the entire experiment. (TIF) [file pone.0067426.s001.tif]
