# Supplementary material for: Characterization of the Link between Ornithine, Arginine, Polyamine and Siderophore Metabolism in Aspergillus fumigatus
Source: PLoS One. 2013 Jun 18;8(6):e67426. doi: 10.1371/journal.pone.0067426 (PMC3688985; doi:10.1371/journal.pone.0067426)
Supplement: Table S1 — Fungal strains used in this study. (DOC) [file pone.0067426.s002.doc]

| **Strain** | **Description** | **Reference** |
| --- | --- | --- |
| ***∆akuB* (KU80)** | *Cea17, akuB* (Afu2g02620)::*pyrG;* | Da Silva Ferreira et al. (2006) |
| **Af293** | *A. fumigatus* clinical isolate | Osherov et al. (2001) |
| **D141** | *A. fumigatus* clinical isolate | Reichard et al. (1990) |
| **Cea10 (CBS 14489)** | *A. fumigatus* clinical isolate | D’Enfert et al. (1996) |
| ***ΔargEF*** | *argEF* (AFUA_6g02910)*::ptrA; akuB* | This study |
| ***ΔargEF^c^*** | *argEF::ptrA; akuB; argEF* | This study |
| ***ΔargB*** | *argB (*AFUA_4g07190*)::pyrG; Af293* | Jadoun et al. (2004) |
| ***ΔargB^c^*** | *argB::pyrG; Af293, argB* | Jadoun et al. (2004) |
| ***ΔcpcA*** | *cpcA* (AFUA_4g12470)*::phleo; D141* | Krappmann et al. (2004) |
| ***ΔsrbA*** | *srbA (*AFUA_2g01260*)::pyrG; Cea17* | Willger et al. (2008) |

**Table S1: Fungal strains used in this study.**
