# Supplementary material for: Characterization of the Link between Ornithine, Arginine, Polyamine and Siderophore Metabolism in Aspergillus fumigatus
Source: PLoS One. 2013 Jun 18;8(6):e67426. doi: 10.1371/journal.pone.0067426 (PMC3688985; doi:10.1371/journal.pone.0067426)
Supplement: Table S2 — Primers used for generation of ΔargEF and argEFc . (DOC) [file pone.0067426.s003.doc]

**Table S2. Primer used for generation of *ΔargEF and argEF^c^.***

| **Primer** | **Sequence 5’-3’** |
| --- | --- |
| **oAfargEF-1** | GGT CTA TTT GCG ATG AGG TTG TG |
| **oAfargEF-2** | CAC GTT GCT GTC TGG TTC |
| **oAfargEF-3** | CAC GTT GCT GTC TGG TTC |
| **oAfargEF-4** | GTC GAG CTC CGA ACG CAG TGT CTA GAG |
| **oAfargEF-5** | CTG GGC GTC AAC AAC TTC |
| **oAfargEF-6** | TCG GAT GAT GCT CAT GCC |
