# Supplementary material for: Characterization of the Link between Ornithine, Arginine, Polyamine and Siderophore Metabolism in Aspergillus fumigatus
Source: PLoS One. 2013 Jun 18;8(6):e67426. doi: 10.1371/journal.pone.0067426 (PMC3688985; doi:10.1371/journal.pone.0067426)
Supplement: Table S3 — Primers used for amplification of hybridization probes. (DOC) [file pone.0067426.s004.doc]

**Table S3: Primers used for amplification of hybridization probes.**

| **Primer** | **gene** | **Sequence 5’-3’** |
| --- | --- | --- |
| **oAfacGS-f** | **acetyl glutamate synthase** | GGA TTG TTC TTG CGG CTG |
| **oAfacGS-r** |  | CCG TCG CAG ATA ACA ACC |
| **oAfamino-f** | **acetyl ornithine aminotransferase** | GAT TTC AGT CGT CGG TCC |
| **oAfamino-r** |  | AGC CGG TTC GAG ATA GAC |
| **oAfArgJ-f** | **arginine biosynthesis bifunctional protein** | AAC TAC TCT GCC CCT CTG |
| **oAfArgJ-r** |  | GTT TGT GCT TGT GTC GCC |
| **oAfCPS-f** | **carbamoyl phosphate synthase** | GCG AGC AGA TCA ACC AAC |
| **oAfCPS-r** |  | CTG ACC GAT CCT TCC TAC |
| **oAfAmcA-f** | **mitochondrial ornithine carrier AmcA** | TCA ATG GAG CTG CCT GTC |
| **oAfAmcA-r** |  | CAA TTC CGT AGC CCT TCG |
| **oAfArg-f** | **arginase** | ACC GCA CCA AAG AGC AAC |
| **oAfArg-r** |  | CGA CGG AGG AAG GAA ATC |
| **oAfdCarb-f** | **ornithine decarboxylase** | AAA GTA CAG CCA GTC GCC |
| **oAfdCarb-r** |  | AAT CAT GGA GGG GAC GAC |
| **oAfArg5,6-f** | **acetylglutamate kinase** | TTC GTA CTC GCC ATA GCC |
| **oAfArg5,6-r** |  | CTT CTC AAT GCT CAC CCC |
| **oAfmirB1me** | **siderophore transporter mirB** | AAG CCG AGA AAA AGG GGG |
| **oAfmirB2me** |  | AAC CCA GAT GAA GCC CAG |
| **oAfArg5,6-f** | **acetylglutamate kinase** | TTC GTA CTC GCC ATA GCC |
| **oAfArg5,6-r** |  | CTT CTC AAT GCT CAC CCC |
| **osidA1** | **L-ornithine monooxygenase SidA** | AAC TAC CTC CAC CAG AAG |
| **osidA2** |  | GAA CGG CAA TGT TGT AAG |
