# Supplementary material for: Characterization of the Link between Ornithine, Arginine, Polyamine and Siderophore Metabolism in Aspergillus fumigatus
Source: PLoS One. 2013 Jun 18;8(6):e67426. doi: 10.1371/journal.pone.0067426 (PMC3688985; doi:10.1371/journal.pone.0067426)
Supplement: Table S4 — Free amino acid pools of ΔargEF ΔargB , ΔcpcA and the respective wt strains. Individual amino acid pools are given in % of the total free amino acids. (DOC) [file pone.0067426.s005.doc]

**Table S4. Free amino acid pools of *ΔargEF* *ΔargB,* *ΔcpcA* the respective wt strains.**

Individual amino acid pools are given in % of the total free amino acids.

|  | 5mM arginine | | 1mM arginine | | 1mM arginine | | 0.2mM arginine | |  |  |
| --- | --- | --- | --- | --- | --- | --- | --- | --- | --- | --- |
|  | ***ΔargEF*** | ***ΔakuB*** | ***ΔargEF*** | ***ΔakuB*** | ***ΔargB*** | **Af293** | ***ΔargB*** | **Af293** | ***ΔcpcA*** | **D141** |
| **Ala** | 4.71±1.23 | 5.48±0.13 | 4.83±0.55 | 5.35±0.10 | 18.55±1.12 | 14.26±1.04 | 21.40±0.55 | 14.16±0.96 | 7.56±0.52 | 14.51±1.02 |
| **Arg** | 24.15±1.69 | 25.65±0.86 | 17.78±1.04 | 18.37±0.93 | 14.77±1.32 | 13.50±0.93 | 5.65±0.91 | 10.03±0.74 | 13.01±0.53 | 13.95±0.32 |
| **Asn** | 2.64±0.23 | 2.69±0.14 | 2.88±0.01 | 2.89±0.32 | 3.54±0.26 | 3.81±0.21 | 4.86±0.48 | 3.95±0.12 | 2.17±0.09 | 2.66±0.09 |
| **Asp** | 4.14±0.04 | 4.26±0.38 | 4.61±0.14 | 5.39±0.24 | 3.31±0.14 | 4.35±0.11 | 3.98±0.06 | 3.46±0.07 | 1.91±0.64 | 2.60±0.41 |
| **Gln** | 42.5±2.12 | 41.41±1.89 | 45.03±0.11 | 45.50±1.42 | 36.02±0.19 | 39.40±1.46 | 34.46±2.55 | 45.43±1.93 | 50.47±0.91 | 33.78±6.69 |
| **Glu** | 15.46±1.92 | 12.77±1.03 | 17.53±0.47 | 13.92±0.66 | 10.63±1.26 | 11.79±0.40 | 11.67±0.16 | 10.72±0.32 | 12.10±0.86 | 14.35±8.06 |
| **Gly** | 0.66±0.13 | 0.96±0.08 | 0.66±0.00 | 1.11±0.04 | 1.51±0.15 | 1.31±0.10 | 1.81±0.43 | 1.33±0.06 | 1.17±0.09 | 1.29±0.10 |
| **His** | 0.74±0.17 | 0.93±0.34 | 1.19±0.03 | 0.88±0.02 | 1.74±0.16 | 1.63±0.08 | 2.25±0.22 | 1.64±0.12 | 1.67±0.17 | 1.86±0.30 |
| **Ile** | 0.17±0.01 | 0.18±0.02 | 0.18±0.01 | 0.20±0.01 | 0.55±0.01 | 0.43±0.02 | 0.79±0.14 | 0.41±0.01 | 0.43±0.02 | 0.62±0.02 |
| **Leu** | 0.16±0.02 | 0.24±0.00 | 0.16±0.01 | 0.27±0.00 | 0.71±0.00 | 0.62±0.02 | 1.03±0.16 | 0.60±0.01 | 0.52±0.09 | 0.90±0.12 |
| **Lys** | 1.8±0.16 | 2.35±0.12 | 2.02±0.01 | 2.79±0.23 | 2.34±0.43 | 2.93±0.62 | 2.83±0.21 | 2.57±0.22 | 4.42±0.61 | 6.62±0.03 |
| **Met** | 0.02±0.01 | 0.03±0.00 | 0.03±0.00 | 0.03±0.00 | 0.11±0.00 | 0.13±0.00 | 0.19±0.01 | 0.12±0.00 | 0.17±0.05 | 0.20±0.02 |
| **Orn** | 0.38±0.02 | 2.46±0.10 | 0.07±0.01 | 2.99±0.07 | 1.66±0.01 | 1.07±0.13 | 5.12±0.76 | 1.64±0.19 | 2.48±0.55 | 5.54±2.53 |
| **Phe** | 0.08±0.01 | 0.09±0.00 | 0.08±0.00 | 0,11±0.01 | 0.27±0.01 | 0.22±0.00 | 0.42±0.08 | 0.21±0.04 | 0.14±0.02 | 0.25±0.02 |
| **Ser** | 1.17±0.11 | 1.18±0.04 | 1.29±0.02 | 1.32±0.02 | 1.76±0.04 | 1.92±0.21 | 2.56±0.21 | 1.92±0.26 | 1.64±0.23 | 2.01±0.10 |
| **Thr** | 1.08±0.12 | 1.09±0.11 | 1.21±0.02 | 1.13±0.02 | 2.09±0.10 | 2.11±0.32 | 2.80±0.43 | 1.89±0.21 | 1.20±0.02 | 1.97±0.32 |
| **Trp** | 0.02±0.00 | 0.03±0.01 | 0.01±0.00 | 0.03±0.00 | 0.17±0.01 | 0.13±0.01 | 0.19±0.00 | 0.12±0.00 | 0.02±0.03 | 0.05±0.01 |
| **Tyr** | 0.16±0.01 | 0.20±0.02 | 0.15±0.00 | 0.24±0.01 | 0.41±0.04 | 0.36±0.01 | 0.81±0.26 | 0.31±0.02 | 0.29±0.03 | 0.49±0.04 |
| **Val** | 0.35±0.05 | 0.45±0.07 | 0.37±0.02 | 0.48±0.01 | 1.55±0.06 | 1.10±0.12 | 2.31±0.19 | 1.15±0.10 | 1.12±0.01 | 1.90±0.21 |
